# Quantitative evaluation of patient setup uncertainty of stereotactic radiotherapy with the frameless 6D ExacTrac system using statistical modeling

**DOI:** 10.1120/jacmp.v17i3.5959

**Published:** 2016-05-08

**Authors:** Vance Keeling, Sabbir Hossain, Hosang Jin, Ozer Algan, Salahuddin Ahmad, Imad Ali

**Affiliations:** ^1^ Department of Radiation Oncology Stephenson Oklahoma Cancer Center, University of Oklahoma Health Sciences Center Oklahoma City OK 73104 USA

**Keywords:** uncertainty model, systemic, random, cumulative uncertainty, stereotactic radiation therapy, frameless

## Abstract

The purpose of this study is to evaluate patient setup accuracy and quantify individual and cumulative positioning uncertainties associated with different hardware and software components of the stereotactic radiotherapy (SRS/SRT) with the frameless 6D ExacTrac system. A statistical model is used to evaluate positioning uncertainties of the different components of SRS/SRT treatment with the Brainlab 6D ExacTrac system using the positioning shifts of 35 patients having cranial lesions. All these patients are immobilized with rigid head‐and‐neck masks, simulated with Brainlab localizer and planned with iPlan treatment planning system. Stereoscopic X‐ray images (XC) are acquired and registered to corresponding digitally reconstructed radiographs using bony‐anatomy matching to calculate 6D translational and rotational shifts. When the shifts are within tolerance (0.7 mm and 1°), treatment is initiated. Otherwise corrections are applied and additional X‐rays (XV) are acquired to verify that patient position is within tolerance. The uncertainties from the mask, localizer, IR ‐frame, X‐ray imaging, MV, and kV isocentricity are quantified individually. Mask uncertainty (translational: lateral, longitudinal, vertical; rotational: pitch, roll, yaw) is the largest and varies with patients in the range $\left(-2.07-3.71\text{mm},-5.82-5.62\text{mm},-5.84-3.61\text{mm};-2.10-2.40^{\circ },-2.23-2.60^{\circ },\text{and}-2.7-3.00^{\circ }\right)$ obtained from mean of XC shifts for each patient. Setup uncertainty in IR positioning (0.88, 2.12, 1.40 mm, and 0.64°, 0.83°, 0.96°) is extracted from standard deviation of XC. Systematic uncertainties of the frame (0.18, 0.25, −1.27mm, $-0.32^{\circ }$, 0.18°, and 0.47°) and localizer (−0.03, −0.01, 0.03 mm, and $-0.03^{\circ }$, 0.00°, $-0.01^{\circ }$) are extracted from means of all XV setups and mean of all XC distributions, respectively. Uncertainties in isocentricity of the MV radiotherapy machine are (0.27, 0.24, 0.34 mm) and kV imager (0.15, −0.4, 0.21 mm). A statistical model is developed to evaluate the individual and cumulative systematic and random positioning uncertainties induced by the different hardware and software components of the 6D ExacTrac system. The uncertainties from the mask, localizer, IR frame, X‐ray imaging, couch, MV linac, and kV imager isocentricity are quantified using statistical modeling.

PACS number(s): 87.56.B‐, 87.59.B‐

## I. INTRODUCTION

The use of stereotactic radiosurgery or stereotactic radiotherapy (SRS/SRT) for treatment of intracranial lesions in 1–5 fractions has been a common modality of treatment for many years.^1^, ^2^ In SRS/SRT treatments with high dose per fraction, it is critical to have accurate localization, effective and reproducible immobilization, and steep dose falloff around the tumor in order to achieve local tumor control and spare normal tissue. Several stereotactic systems have been employed by different vendors to accurately immobilize these patients during the treatment.^3^, ^4^, ^5^, ^6^ However these SRS/SRT treatments usually involve invasive immobilization, such as metal frames or rings fixed to the patient's skull, which provides rigid immobilization and reproducible stereotactic coordinate system.^1^, ^7^ These invasive immobilization devices are uncomfortable for the patient and require more time and manpower to simulate, plan, and deliver the treatment on the same day. Frameless stereotactic radiotherapy systems have become a popular treatment option for intracranial radiosurgery or radiotherapy due to less invasive patient immobilization, along with high accuracy in image guidance.^8^, ^9^, ^10^, ^11^, ^12^, ^13^

The integration of image‐guided radiotherapy tools, such as MV^14^, ^15^ and kV^16^, ^17^ on‐board imaging (OBI) mounted on linear accelerators, have increased efficiency and reproducibility of patient setup and target positioning. The kV OBI systems provide diagnostic quality images of a patient's internal anatomy with high positional accuracy and high contrast.^(18)^ Images are usually obtained at two orthogonal angles and compared to their corresponding digitally reconstructed radiographs (DRRs) generated by the treatment planning system (TPS) using simulation CT images. Image fusion algorithms mostly rigid are then employed to match these images based on bone anatomy. Shifts are then calculated and applied by moving the treatment couch to a position that matches anatomically with the reference images.

In this work, the uncertainty of patient positioning and tumor localization using the frameless 6D Brainlab ExacTrac system (Brainlab, Inc, Westchester, IL) is investigated. This system uses a noninvasive thermoplastic immobilization mask and stereotactic localizer which increases patient comfort and allows faster patient setup and fractionated treatment as compared to invasive immobilization systems. Accurate patient positioning and dose delivery to the target are achieved by image guidance combined with noninvasive immobilization. This system uses a combination of infrared (IR) tracking and kV stereoscopic imaging for accurate patient setup. In a previous work,^(19)^ an evaluation of setup uncertainty is determined for both the IR system and KV imaging system. In this study, the uncertainties associated with the different hardware and software components employed in the stereotactic treatment process with the Brainlab system are investigated by measurement and modeling. A statistical model is developed to investigate the individual and cumulative uncertainties of the different components used by the 6D ExacTrac system from Brainlab.

## II. MATERIALS AND METHODS

### A. Brainlab immobilization and localization

**Figure 1:**
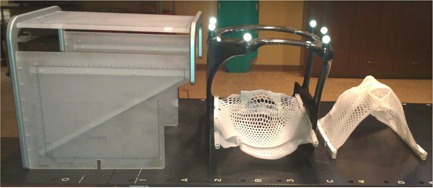
(left) CT localizer, (middle) infrared frame, (middle and right) lower and upper components of the head and neck Brainlab mask.

Patient immobilization for intracranial SRS/SRT is accomplished with a noninvasive thermoplastic mask, as shown in Fig. 1. The thermoplastic mask system is made from three different layers. One layer is molded to the back of the patient's head. A second layer contains three reinforcing straps, and a third layer is placed over the mask with three straps which covers the shoulders and face. These masks are attached to a couch‐mounted support system which provides rigid fixation of the patients head. The 2 mm spacers are initially used in making the patient mask and CT simulation which allow for other spacers with different sizes to replace it in case the patient's mask becomes tight or loose during the course of treatment. The patients are imaged with a localizer box that contains 6 rods made from dense material that shows high contrast on the simulation CT images. Three rods in different orthogonal planes have the same position along the superior–inferior direction in all CT slices, and another three diagonal rods change position in each CT slice. The treatment planning system detects the position of the rods on the CT images and employs them to define a reproducible and precise stereotactic reference coordinate system.

### B. Infrared positioning and X‐ray imaging with ExacTrac system

**Figure 2:**
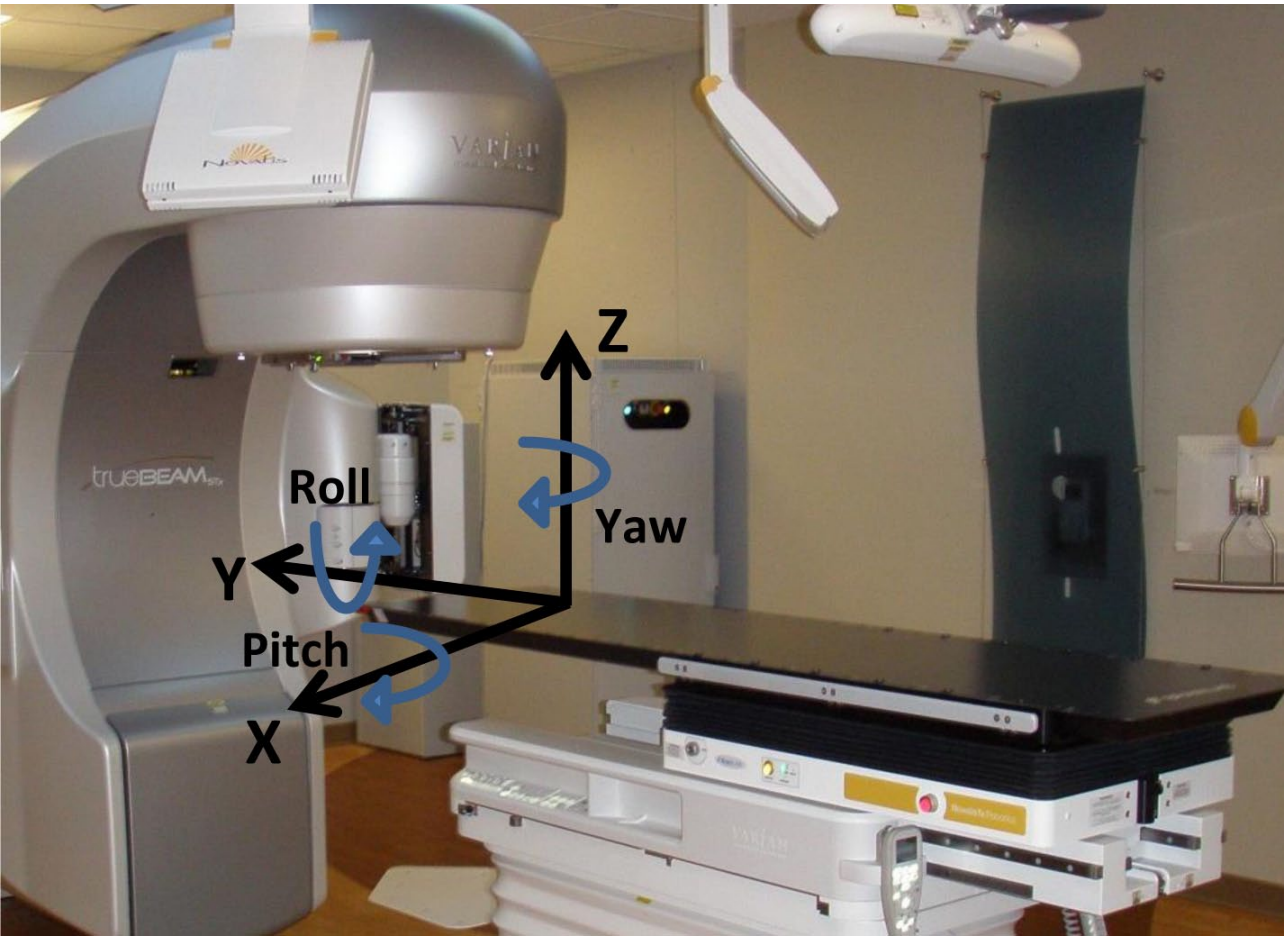
Schematic of patient/couch coordinates system (X, Y, Z) where the lateral shifts are along x‐axis, superior‐inferior shifts are along the y‐axis, and anterior‐posterior shifts are along the z‐axis. The pitch rotation is around x‐axis, roll is around y‐axis, and yaw is around z‐axis.

Thirty‐five patients (sometimes with multiple isocenters, 49 treatments in total) are treated with a frameless thermoplastic mask in 1, 3, or 5 fractions with image guidance using the ExacTrac system. The patient setup is performed with infrared (IR) optical imaging and stereoscopic kV X‐ray imaging. First, stereotactic patients are set up to the isocenter position using IR guidance. The IR detects infrared markers on the cranial frame that correlate the position of the markers with the isocenter position generated by the treatment planning system using the stereotactic localizer used in the CT simulation images. Second, two radiographic images (X‐ray correction, XC) are acquired and matched with reference DRRs from the simulation CT images that are generated using ExacTrac software. Matching of DRR and radiographic image is performed with rigid image fusion using bone‐anatomy matching. This fusion generates translational and rotational 6D couch shifts used for patient setup, as shown in Fig. 2. If the shifts are below our institutional criteria, within 0.7 mm and 1°, then the treatment is initiated. Otherwise shifts are applied by moving a robotic couch that is capable of 6D translational and rotational shift correction. After application of the couch shifts, a second set of radiographic images (X‐ray verification: XV) are acquired to verify patient position based on internal anatomy and that final patient translational and angular positions are within a tolerance of 0.7 mm and 1°, respectively. This process is repeated until all the shifts based on patient anatomical matching with X‐ray imaging are within tolerance.

Quality assurance (QA) of the Brainlab system and the dose delivery machine is performed daily before initiation of Brainlab SRS/SRT treatments. It includes both machine and patient‐specific QA procedures. Isocentricity of the dose delivery MV machine and different kV imaging systems are checked to verify that all isocenters, including lasers, light, radiation, IR, and X‐ray, coincide within 1 mm. The isocentricity check of the MV beam is performed with a Winston‐Lutz pointer phantom (Brainlab) that contains a lead ball which (5 mm) is aligned to room laser. The IR isocentricity is tested with the isocenter calibration phantom that possesses infrared markers and crosshairs that align automatically by the ExacTrac software to the IR imaging isocenter matching the lasers which, in turn, match with radiation isocenter. Two radiographs from the ExacTrac imaging system are taken of the isocenter pointer phantom which contains a high contrast ball inside to test isocentricity of the X‐ray imager.

### C. Patient setup shifts and data analysis

XC and XV 6D translational and rotational shifts are collected in a database for each patient with all treatment fractions. For each patient, the mean and standard deviation (σ) of translational and rotational shifts are calculated for XC and XV for all fractions. If a patient has only one fraction, then this single data point is used as the mean and zero mm standard deviation. Histograms of the mean and σ are generated for XC and XV 6D‐shifts (x (Lat), y (Long), z (Vert), ρ (Pitch), θ (Roll), φ (Yaw)), as shown in Fig. 2. The radial uncertainty is calculated by quadrature sum of the translational components in three directions $\Sigma r=\sqrt{\left(\Sigma_{x}^{2}+\Sigma_{y}^{2}+\Sigma_{z}^{2}\right)}$ for each fraction. Statistical significance of the means of the shifts in each direction is determined using the Student's *t*‐test with values ≥0.05, indicating statistically insignificant differences in the 95% confidence level.

### D. Uncertainty modeling for the frameless ExacTrac Brainlab system

SRS/SRT treatment with Brainlab system is composed from several hardware and software components that introduce patient positioning or tumor localization uncertainties in the different processes including: a) patient immobilization and CT simulation, b) stereotactic localization and treatment planning, c) patient setup and dose delivery, d) isocentricity of the radiation, optical, and X‐ray imaging systems. The positioning uncertainties of the previous processes are quantified by a statistical model which includes the main software and hardware components. If the cumulative positioning function of the frameless ExacTrac Brainlab system is given by $P_{c}$, then it depends on the different patient setup processes that occur sequentially and interdependently on each other as follows:

(1) $$P_{c}(x,y,z,\rho ,\theta ,\varphi )=P(M,L,F,XR,C,ISO_{MV},ISO_{KV},ISO_{IR})$$

where M(x,y,z,ρ,θ,ϕ) is a positioning function that represents the immobilization mask and depends on 6D translational and rotational shifts, L(x,y,z,ρ,θ,ϕ) is the localizer function employed to define the stereotactic reference frame, and F(x,y,z,ρ,θ,ϕ)) is the positioning function associated with patient setup with the infrared system. XR(x,y,z,ρ,θ,ϕ) is the positioning function of the patient with X‐ray radiographs obtained from ExacTrac that are registered with reference DRRs using bone‐anatomy matching. C(x,y,z,ρ,θ,ϕ) is the couch positioning function associated with sagging effects. $ISO_{MV}(x,y,z)$ is a function that measures the position offset of isocenter of the radiotherapy MV machine from the mechanical isocenter which is obtained by a combined offset from the rotation of the gantry, collimator, and table. $ISO_{kV}(x,y,z)$ represents the isocentricity of the kV imager. $ISO_{IY}(x,y,z)$ is the isocentricity of IR system.

Each of the previous processes is associated with uncertainties that propagate from one step into another through the patient simulation, planning, setup, and treatment. The cumulative uncertainty $\left(\Delta P_{c}\right)$ is calculated by taking the partial derivatives of Eq. (1) as follows:

(2) $$\begin{array}{lll} \Delta P_{C} & = & \frac{\partial P_{C}}{\partial M}\Delta M+\frac{\partial P_{C}}{\partial L}\Delta L+\frac{\partial P_{C}}{\partial F}\Delta F+\frac{\partial P_{C}}{\partial XF}\Delta XR+\\ & & \frac{\partial P_{C}}{\partial ISO_{MV}}\Delta ISO_{MV}+\frac{\partial P_{C}}{\partial ISO_{kV}}\Delta ISO_{kV}+\frac{\partial P_{C}}{\partial ISO_{IR}}\Delta ISO_{IR} \end{array}$$

where $\left(\Delta M,\Delta L,\Delta F,\Delta XR,\Delta C,\Delta ISO_{MV},\Delta ISO_{kV},\Delta ISO_{IR}\right)$ are the positioning uncertainties associated with the mask, localizer, frame, couch, and MV radiotherapy, kV imaging and IR system isocentricity, respectively. For example, the mask provides noninvasive immobilization that induces uncertainties from one treatment session into another in the translational and rotational directions as follows:

(3) $$\Delta M=\frac{\partial M}{\partial x}\Delta x+\frac{\partial M}{\partial y}\Delta y+\frac{\partial M}{\partial z}\Delta z+\frac{\partial M}{\partial \rho }\Delta \rho +\frac{\partial M}{\partial \theta }\Delta \theta +\frac{\partial M}{\partial \varphi }\Delta \varphi$$

where Δx,Δy,Δz,Δρ,Δθ,Δϕ are uncertainties resulting from 6D translational and rotational shifts.

The uncertainties from the different software and hardware components of the Brainlab system can be systematic or random which propagate from one step to other sequential steps through the treatment process. The uncertainty distributions are fitted with normal distribution functions where systematic uncertainties are obtained from the mean values, while random uncertainties are obtained from the standard deviations. Considering independent processes, the cumulative systematic uncertainties $\left(\Sigma_{P}\right)$ are obtained from addition of the mean of the normal distribution of the different processes as follows:

(4) $$\sum_{P}=\sum_{M}+\sum_{L}+\sum_{F}+\sum_{XR}+\sum_{ISO_{MV}}+\sum_{ISO_{kV}}+\sum_{ISO_{IR}}$$

The cumulative random uncertainties are obtained by quadrature addition of the standard deviation of the different processes as given in the following:

(5) $$\sigma_{P}={\left\{\sigma_{M}^{2}+\sigma_{L}^{2}+\sigma_{F}^{2}+\sigma_{XR}^{2}+\sigma_{ISO_{MV}}^{2}+\sigma_{ISO_{kV}}^{2}+\sigma_{ISO_{IR}}^{2}\right\}}^{1/2}$$

Although the mask system provides reproducible noninvasive immobilization, it is less rigid than immobilization with the halo ring. The mask setup varies from one patient to another, depending on the preparation and manufacturing quality of the mask, and this introduces systematic uncertainty $\left(\Sigma_{M}\right)$ associated with each patient treated with a specific mask. In this model, the couch systematic uncertainty is measured by the mean of the normal distribution along the different directions and the corresponding cumulative value is obtained by simple addition of the systematic uncertainties in the different directions. Each SRS/SRT patient is set up with the mask before each treatment session which introduces random uncertainty $\left(\sigma_{S}\right)$ in the patient treatment from one sessions into another. $\sigma_{S}$ is obtained from the standard deviation of the normal distributions. The cumulative random uncertainty is obtained by quadrature sum of the random uncertainties in the different directions.

The treatment planning system uses a localization frame which is imaged with the patient in CT simulation to create the stereotactic reference frame. The treatment isocenter position is calculated in this reference frame. Systematic uncertainties in the calculation of isocenter position $\left(\Sigma_{L}\right)$ are induced because of the limited accuracy of the localization process that is determined by various factors including the detection efficiency and positioning of the localizer fiducial markers on the CT images with certain slice thickness (1.25 mm). The patients are set up to the treatment position with IR imaging which detects the markers on the IR positioning frame. The frame introduces systematic uncertainty $\left(\Sigma_{F}\right)$ that depends on the accuracy and precision of the frame and efficiency of the infrared marker detection algorithm. After patient positioning with IR, radiographic images are acquired where the patient is set up based on bone‐anatomy matching with the reference DRRs from simulation CT images. Systematic uncertainty $\left(\Sigma_{\text{XR}}\right)$ is introduced due to limitations of the image quality acquired by the ExacTrac system and DRRs employed by the image registration algorithm. The X‐ray imaging corrects random uncertainty in the patient setup $\left(\sigma_{S}\right)$ due to inaccurate patient positioning within the mask. Couch sagging introduces systematic positioning uncertainty $\left(\Sigma_{C}\right)$ in the different directions and angles that depends on the accuracy of the bearing system and patient weight. The uncertainty in sagging of the couch $\left(\Sigma_{C}\right)$ is determined with a phantom study by placing varying amounts of weight (0, 10, 50, and 70 kg) on the Brainlab ExacTrac couch at various couch angles (0°, 45°, 90°, 270°, and 315°) and using the IR system to determine the sag. Other systematic uncertainties are introduced by isocentricity of the radiotherapy machine $\left(\Sigma_{{\text{ISO}}_{\text{MV}}}\right)$ which results from the mobile components that include gantry, collimator, and couch rotations. These uncertainties result from the mismatch of the rotation isocenter of the gantry, collimator, and couch, and the radiation isocenter of the machine as given by the following equation:

(6) $$\begin{array}{l} \sum_{ISO_{MV}}=\sum_{G}+\sum_{Coll}+\sum_{T} \end{array}$$

where $\Sigma_{G}$ is the systematic uncertainty associated with gantry rotation, $\Sigma_{Coll}$ is systematic uncertainty associated with collimator, and $\Sigma_{T}$ is associated with couch isocentricity, which is different from couch sag due to patient weight.

Similarly, the kV ExacTrac imaging system contributes with positioning uncertainties in patient setup due to mismatch with the radiation isocenter and actual mechanical isocenter. Similarly, the isocentricity of the ExacTrac imaging system introduces further systematic uncertainty $\left(\Sigma_{{\text{ISO}}_{\text{kV}}}\right)$ in patient setup due to mismatch with the actual mechanical isocenter. The positioning with the ExacTrac IR system is also associated with systematic uncertainty $\left(\Sigma_{{\text{ISO}}_{\text{IR}}}\right)$ which measures mismatch of the isocenter of the IR system with the radiation isocenter. The translational offsets of the MV, kV, and IR isocenters were measured using the Winston‐Lutz test from the monthly quality assurance procedure at our institution for nearly two years. The individual uncertainties associated with the ExacTrac system are listed in Table 1.

The cumulative uncertainty of the SRS/SRT treatment with the frameless Brainlab ExacTrac system $\left(\Delta_{\text{PC}}\right)$ considering the previous different positioning uncertainties is obtained in two ways: a) the summation of systematic and random uncertainty for the different components, as given in Eq. (7) where systematic uncertainties are added linearly and random uncertainties are added by quadrature, and b) using the GUM standard uncertainty(^20‐22)^ with quadrature summation of systematic and random uncertainties as given by Eq. (8).

(7) $$\begin{array}{l} \sum_{P}=\sum_{M}+\sum_{L}+\sum_{F}+\sum_{XR}+\sum_{C}+\sum_{ISO_{MV}}+\sum_{ISO_{kV}}+\sum_{ISO_{IR}}\\ \sigma_{P}={\left(\sigma_{S}^{2}+\sigma_{XR}^{2}\right)}^{1/2}\\ \Delta_{P_{C}}=\sum_{P}+\sigma_{P} \end{array}$$

(8) $$\sum_{P}^{GUM}={\left(\sum_{M}^{2}+\sum_{L}^{2}+\sum_{F}^{2}+\sum_{XR}^{2}+\sum_{C}^{2}+\sum_{ISO_{MV}}^{2}+\sum_{ISO_{kV}}^{2}+\sum_{ISO_{IR}}^{2}+\sigma_{S}^{2}+\sigma_{XR}^{2}\right)}^{1/2}$$

**Table 1:** Positioning uncertainty model for the SRT/SRS treatment with the frameless ExacTrac Brainlab system.

$\Delta_{M}$	Mask systematic uncertainty	Mean of shifts from the XC data for each patient
$\sigma_{S}$	Setup random uncertainty	Standard deviation of the XC data for each patients
$\Delta_{F}$	IR frame systematic uncertainty	Mean of the XC data for all patients
$\Delta_{L}$	Localizer systematic uncertainty	Mean of XV data
$\sigma_{\text{XR}}$	X‐ray image quality and image registration algorithm	Standard deviation of means of XV data
$\Delta_{C}$	Couch sagging	Phantom study with various couch angles and weights
$\Delta_{{\text{ISO}}_{\text{MV}}}$	Radiation isocenter	Machine Winston‐Lutz QA data
$\Delta_{{\text{ISO}}_{\text{kV}}}$	Imaging isocenter	Machine Winston‐Lutz QA data
$\Delta_{{\text{ISO}}_{\text{IR}}}$	IR optical imaging isocenter	Machine Winston‐Lutz QA data

## III. RESULTS

The results are represented in the same order as the uncertainties from the model for the different software and hardware components as represented previously in the Material and Methods section. The XC shift distributions in Results section A are used to extract the mask and frame systematic uncertainties and patients random uncertainties due to daily variations in patient setup. The mean of the XV distribution in Results B provides the systematic uncertainty for the localizer; the standard deviations represent random uncertainty due X‐ray image quality variation and image registration. Then, the systematic uncertainties for the isocenters of the kV, MV, and IR in Results C are represented, followed by uncertainty data due to couch sagging in Results D. In the last section, Results E, the cumulative uncertainties calculated from this statistical model and GUM method are compared.

### A. X‐ray correction

**Figure 3:**
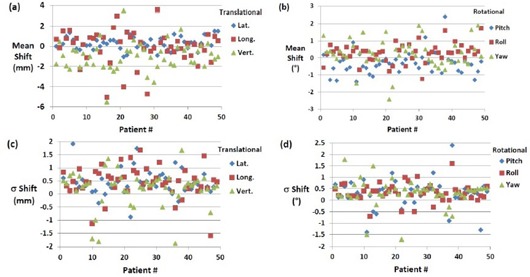
Mean of translational shifts (a) (lateral, longitudinal, and vertical) and (b) mean of rotational shifts (roll, pitch, and yow) for all 49 patient treatments from XC setup. Standard deviations of the translational (c) and rotational (d) XC shifts for all 49 patient treatments.

Figure 3 shows the mean 6D translational and rotational shifts for each patient from XC setup. In most cases, the mean translational shifts are from −2.5 to 2.5 mm, while rotational shifts are usually less than 1°. Uncertainties as large as 5.84 mm and 2.4° for translational and rotational shifts are found, respectively. These outliers represent masks where patients do not setup appropriately because of the limited manufacturing quality of the mask, loosening or shrinkage of the mask during treatment or the physical changes of the patient's head during treatment where some patients have swelling of the face due to administration of steroids. The mean shifts represent the systematic uncertainties in the different directions $\left(\Sigma_{M}^{X,Y,Z},\Sigma_{M}^{\rho ,\theta ,\phi }\right)$ for each mask used specifically in a patient treatment. Figures 3(c) and (d) show the σ of the translational and rotational XC shifts for each of the 49 patient treatments which represent random setup uncertainties $\left(\sigma_{S}^{X,Y,Z},\sigma_{S}^{\rho ,\theta ,\phi }\right)$ associated with each patient. Generally the standard deviation is much less than 1 mm and 1° for translational and rotational shifts, respectively. The average σ for all patients is 0.42, 0.46, and 0.20 mm for lateral, longitudinal, and vertical shifts, respectively.

The reproducibility in patient setup is much better in the lateral direction than it is in the longitudinal and vertical direction, as shown in Fig. 3(c). The average σ in the rotational shifts are 0.30°, 0.33°, and 0.32° for pitch, roll, and yaw, respectively. In few cases, IR positioning alone (XC) (16 out of 203 setups) satisfies our setup tolerances and X‐ray verification is not needed.

**Figure 4:**
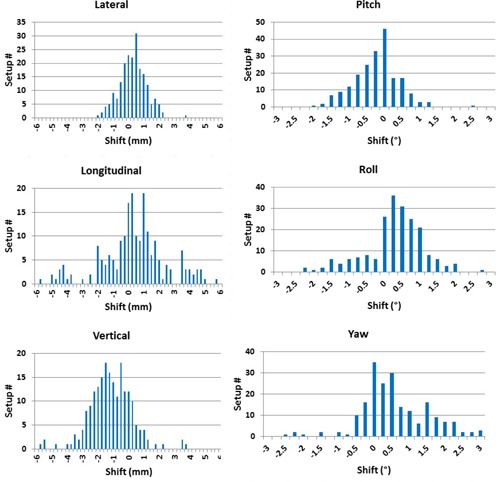
Histograms of translational and rotational shifts for all patient setups (203 setups) obtained from X‐ray correction (XC).

Figure 4 shows histograms of the translational and rotational shifts for all 203 treatment fractions for the 49 patients. The mean of these shifts represents the systematic uncertainties associated with the frame $\left(\Sigma_{F}^{X,Y,Z},\Sigma_{F}^{\rho ,\theta ,\phi }\right)$. Table 2 lists mean, σ, p‐values, minimal (Min), and maximal (Max) translational and rotational X‐ray correction shifts for all 203 treatment fractions. The standard deviation shifts represent the random uncertainties $\left(\sigma_{S}^{X,Y,Z},\sigma_{S}^{\rho ,\theta ,\phi }\right)$ of patient setup with frameless ExacTrac using the HN mask for immobilization, which are listed in Table 2, while the mean of XC shifts represents the systematic uncertainties of the IR frame in the different directions $\left(\Sigma_{F}^{X,Y,Z},\Sigma_{F}^{\rho ,\theta ,\phi }\right)$ which contribute to each patient setup, as listed in Table 2.

**Table 2:** Mean, σ, p‐values, minimal (Min), and maximal (Max) translational and rotational X‐ray correction shifts for all 203 treatment fractions.

	*Translational (mm)*				*Rotational (°)*		
	*Lat.*	*Long. Vert.*	*Quad. Sum*	*Pitch*	*Roll*	*Yaw*
Mean±σ	0.18±0.88	0.25±2.12	−1.27±1.40	2.52±1.60	−0.32±0.64	0.18±0.83	0.47±0.96
p‐value	0.67	0.00	0.00		0.00	0.00	0.00
Min	−2.07	−5.82	−5.84	0.26	−2.10	−2.30	−2.70
Max	3.71	5.62	3.61	8.05	2.40	2.60	3.00

### B. X‐ray verification

**Figure 5:**
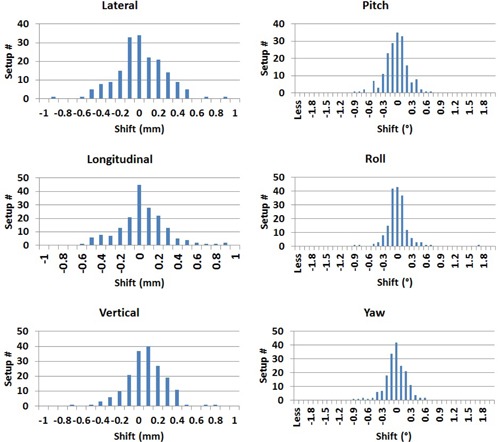
Histograms of the 6D translational and rotational shifts from X‐ray verification (XV).

**Figure 6:**
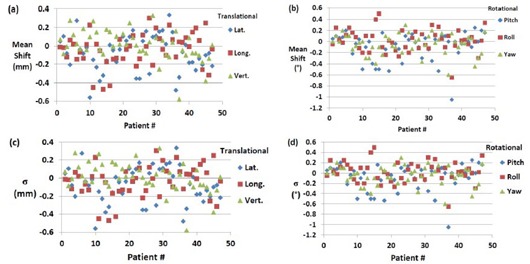
Mean of the translational (a) and rotational (b) XV shifts for each patient. The standard deviations of translation (c) and rotational (d) shifts (XV) for 47 patients.

Figure 5 shows histograms for all 6D translational and rotational XV shifts which are acquired as a verification of patient setup using X‐rays. The range of XV translational and rotational shifts vary depending on direction and angle, as listed in Table 3. Figures 6(a) and (b) show the means of the 6D shifts for each patient treatment from the XV setup. In most cases the uncertainty is less than 0.3 mm and 0.3° for the translational and rotational shifts, respectively. Figures 6(c) and (d) represent σ of the 6D XV shifts for each patient. Table 3 lists the mean and standard deviation for all 6D XV shifts and the corresponding p‐values. On average translation shifts are less than 0.1 mm and less than 0.1° for rotational shifts. The σ of XV shifts is highest in the longitudinal direction. The difference in translational shifts is only significant for lateral and vertical directions. No statistically significant differences are found for the rotational shifts with all comparisons having a p‐value greater than 0.5. The majority of cases only require single X‐ray verification to achieve our institution setup criteria. However, nine setups required a second X‐ray verification to achieve setup tolerance criteria, with some of them requiring patient repositioning within the mask. The mean of all XV shifts gives the systematic uncertainty in the stereotactic localization algorithm $\left(\Sigma_{L}^{X,Y,Z},\Sigma_{L}^{\rho ,\theta ,\phi }\right)$ used by the iPlan treatment planning and ExacTrac systems which depends mainly on CT image quality and slice thickness. These localization systematic uncertainties in the translational and rotational directions are (−0.03mm, −0.01mm, and 0.03 mm, and $-0.03^{\circ }$, 0.02°, and 0.01°), respectively, as listed in Table 3. The standard deviation in XV shifts represents the random uncertainties $\left(\Sigma_{XR}^{X,Y,Z},\Sigma_{XR}^{\rho ,\theta ,\phi }\right)$ induced by the image registration algorithm that matches bone anatomy on the X‐ray images and reference DRRs from ExacTrac. These uncertainties are 0.25 mm, 0.26 mm, 0.21 mm and 0.25°, 0.24°, 0.24°, as listed in Table 3. The random uncertainties result from variations in X‐ray image quality due to variations in imaging beam quality, noise, and scattering radiation induced by the treatment couch, immobilization devices, and other patient setup equipment.

**Table 3:** Mean, standard deviation, p‐value, minimal (Min), and maximal (Max) shifts for 179 X‐ray verification shifts (translational and rotational).

	*Translational (mm)*				*Rotational (°)*		
	*Lat.*	*Long. Vert.*	*Quad. Sum*	*Pitch*	*Roll*	*Yaw*
Mean±σ	−0.03±0.25	−0.01±0.26	0.03±0.21	0.36±0.21	−0.03±0.25	0.00±0.24	−0.01±0.24
p‐value	0.33	0.01	0.17		0.33	0.59	0.60
Min	−0.92	−0.64	−0.77	0.04	−0.90	−0.90	−0.90
Max	0.84	0.87	0.78	1.25	0.70	1.70	0.60

### C. MV, kV, and IR isocentricity

The mean and standard deviation of translational position offsets of the MV $\Sigma_{{ISO}_{MV}}^{X,Y,Z},\sigma_{{ISO}_{MV}}^{X,Y,Z})$ and kV $\Sigma_{{ISO}_{kV}}^{X,Y,Z},\sigma_{{ISO}_{kV}}^{X,Y,Z})$ and IR $\Sigma_{{ISO}_{IR}}^{X,Y,Z},\sigma_{{ISO}_{IR}}^{X,Y,Z})$ isocenters are calculated from the Winston‐Lutz test from measurements acquired from the monthly mechanical quality assurance procedure at our institution for nearly two years. The mean and standard deviation of the position offset for the gantry, collimator, couch for the MV machine, and kV gantry rotation are listed in Table 4. The mean offset of the radiation isocenter is 0.27, 0.24, and 0.34 mm for gantry in the lateral, longitudinal, and vertical directions, respectively.

**Table 4:** Mean and standard deviation (σ) of the position offset for the gantry, collimator, couch rotations for the MV machine, and kV gantry rotation using Winston‐Lutz test.

*Isocenter Measurements* (Mean±σ)			
	*Lat (mm)*	*Long (mm)*	*Vert (mm)*
Gantry Radiation Isocenter	0.27±0.27	0.24±0.24	0.34±0.34
Collimator Radiation Isocenter	0.32±0.21	0.24±0.14	n/a
Couch Radiation Isocenter	0.32±0.21	0.22±0.13	n/a
X‐ray	0.15±0.80	−0.40±0.16	0.21±0.09
Infrared Isocenter	0.02±0.01	0.02±0.01	0.02±0.01

### D. Couch sag

Couch sagging shifts the patient alignment from radiation isocenter which leads to small translational and rotational uncertainties, as shown in Table 5. It lists the translational and rotational uncertainties due to sagging of the robotic treatment couch $\left(\Sigma_{C}^{X,Y,Z},\Sigma_{C}^{\rho ,\theta ,\phi }\right)$ at different couch angles and weights from the phantom study. The couch sag is mostly dependent on the couch angle rather than weight of the phantom. After placement of each weight, the couch position is set to zero translational and rotational shifts at zero degree couch angle. As couch is rotated at angles different from zero, couch sag increases; however, it is not always increasing with increase in weight. For example at 45° couch angle, the quadrature sum of translational uncertainties is 0.70, 0.67, 0.63, and 0.67 mm for 0, 10, 50, 70 kg weights, respectively. At the couch angle of 315°, the quadrature sum of the translational uncertainties steadily increases with increasing weight as follows: 0.78, 0.84, 0.92, 1.09 mm for 0, 10, 50, 70 kg, respectively. Uncertainty in couch sag is greatest at couch angle of 270° and 315° using a weight of 70 kg for this specific couch. The sagging shifts depend on couch angle and patient weight which is corrected usually with IR and X‐ray imaging. However, at certain couch angles, these shifts cannot be corrected because ExacTrac imager is blocked by couch, particularly at 90° and 270°.

**Table 5:** Translational and rotational couch sagging uncertainty of the Brainlab robotic couch for various couch angles and weights.

		*Translational (mm)*				*Rotational (°)*		
*Weight (kg)*	*Couch Angle (°)*	*Lat*	*Long.*	*Vert.*	*Quad. Sum*	*Pitch*	*Roll*	*Yaw*
0	0	0.01	0.01	0.01	0.02	0.30	−0.10	n/a
	45	−0.20	0.60	−0.30	0.70	0.40	−0.10	n/a
	315	0.20	0.04	−0.75	0.78	0.10	0.10	n/a
10	0	0.01	0.01	0.01	0.02	0.20	−0.10	n/a
	45	0.00	0.60	−0.30	0.67	0.30	−0.10	n/a
	315	0.30	0.07	−0.78	0.84	0.00	0.10	n/a
50	0	0.01	0.01	0.01	0.02	0.20	−0.10	n/a
	45	0.25	0.50	−0.30	0.63	0.30	−0.10	n/a
	315	0.48	0.08	−0.78	0.92	0.00	0.10	n/a
70	0	0.01	0.01	0.01	0.02	0.10	−0.10	n/a
	45	0.01	0.64	−0.20	0.67	0.30	−0.10	n/a
	90	−0.25	0.37	−0.10	0.46	0.00	0.10	n/a
	270	0.40	−0.97	−0.29	1.09	0.30	0.00	n/a
	315	0.85	0.01	−0.68	1.09	−0.10	0.20	n/a

### E. Individual and cumulative uncertainty

**Figure 7:**
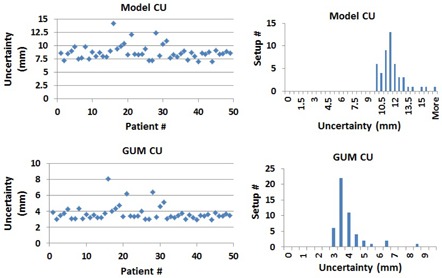
Cumulative uncertainty (CU) of patient setup of the different hardware and software components using the uncertainty model developed in this work (top panel) and the GUM method (bottom panel).

Table 6 lists a summary of the main systematic and random uncertainties from the different hardware and software components of the SRS/SRT Brainlab treatment with ExacTrac system, as determined by this uncertainty model. These uncertainties are employed to calculate a cumulative uncertainty (CU) for each patient before and after X‐ray imaging (XC) of the patient setup using the model developed in this work and the GUM report and AAPM TG‐138.^20^, ^21^, ^22^ The CU before XC represents an evaluation for all combined uncertainties induced by the different components of the SRS/SRT Brainlab system for the different patients, as shown in Fig. 7. Using Eq. (7) for this uncertainty model, the mean ±σ of CU is 11.44 ± 1.40 mm and the range of uncertainties is 9.70–16.86 mm for all patients before XC. The corresponding mean ±σ of CUs determined by GUM using Eq. (8) is 3.73 ± 0.96 mm with a range 2.95–8.04 mm, which is much smaller than the one calculated from this uncertainty model before XC. The CU after XC which remains in treatment of most SRS/SRT patients is 3.29 mm and 1.43 mm for this model and GUM, respectively. The GUM expanded uncertainty after XC is 2.86 mm which is obtained by multiplying the GUM cumulative accuracy with a factor of 2.^20^, ^21^, ^22^ CU after XC is the same uncertainty for all patients because it results from systematic uncertainties that are associated with software and hardware components of the SRS/SRT Brainlab system that are not related to the patient.

**Table 6:** Summary of the individual and cumulative systematic and random uncertainties for the different components of the SRS/SRT treatment with the Brainlab ExacTrac system.

*Uncer.*	*SRS/SRT Equipment*	*Type*	*Lat (mm)*	*Long (mm)*	*Vert (mm)*	*Roll (^o^)*	*Pitch (^o^)*	*Yaw (^o^)*	*Rad (mm)*
$\Delta_{M}$	Mask	Systematic	Depends on the mask manufactured for each patient as shown in Fig. 1.						
$\sigma_{S}$	Setup	Random	Standard Deviation of Mean Quad Sum XC						
$\Delta_{F}$	Frame	Systematic	0.18	0.25 p=0.01	−1.27 p=0.17	0.32 p=0.33	0.18 p=0.59	0.47 p=0.60	2.52
$\Delta_{L}$	Localizer	Systematic	−0.03 p=0.33	−0.01	0.03	−0.03	0.00	−0.01	0.36
$\sigma_{\text{XR}}$	X‐ray	Random	0.20	0.18	0.16	0.26	0.20	0.19	0.16
$\Sigma_{\text{ISO}-\text{MV}}$	Isocenter of MV beam	Systematic	0.27	0.24	0.34	n/a	n/a	n/a	
$\Sigma_{\text{ISO}-\text{KV}}$	Isocenter of kV imager	Systematic	0.15	−0.40	0.21	n/a	n/a	n/a	
$\Sigma_{\text{ISO}-\text{IR}}$	Isocenter of IR system	Systematic	0.02	0.02	0.02	0.01	0.01	0.01	
$\Delta_{C}$	Couch sag	Systematic	0.85	0.01	−0.68	−0.10	0.20	n/a	1.09

## IV. DISCUSSION

Uncertainty in Brainlab treatment setup is dominated by the mask positioning uncertainty. The IR imaging system accurately moves the patient to isocenter based on the positioning of the IR markers which do not necessarily correlate with patient anatomy within the mask. Although the H&N masks from Brainlab are generally tight, it seems that they do not prevent patients from moving or flexing within the mask which results in anatomical mismatch with the patient position in CT simulation used by the treatment planning system. Therefore, patient setup with the infrared system is often not sufficient for accurate positioning and should always be followed up by XC and XV. The XC and XV corrections remove systematic and random uncertainties in patient setup due to patient positioning within the mask with the patient at 0° angle couch position. This approach is justified for a number of reasons including: a) brain anatomy does not change suddenly, b) patient weight loss or motion does not affect patient setup for brain treatments^(3)^ in contrast to extracranial tumors,^(23)^ and c) the shifts are calculated from bone‐anatomy matching, which is very stable and reproducible in SRS/SRT treatments with the Brainlab system.

Couch sagging due to changing the position of the treatment couch to noncoplanar angles induces systematic uncertainty that is dependent on the couch position and patient weight. Couch sagging is corrected by snap verification with one or two X‐ray images that are used to reposition the patient at a particular couch position; however, they are not corrected when the couch is blocking the ExacTrac imaging view. The isocentricities of the MV machine and kV imager induce systematic uncertainties in the patient position that are not corrected by XC or XV. These uncertainties are quantified by the Winston‐Lutz test that is performed before each SRS/SRT treatment with the Brainlab system. Using the uncertainty model developed in this work, cumulative uncertainty from the different hardware and software components are calculated and compared with the GUM or the AAPM Task Group‐138 method.^20^, ^21^, ^22^ The GUM method seems to underestimate the CU before XC and deviates from this model uncertainty by nearly a factor of 3. In this model (Eq. (7)) introduced here, the cumulative uncertainty is added linearly for systematic uncertainties, and then both systematic and random uncertainties are added by quadrature. In the GUM model, all uncertainties are added by quadrature (Eq. (8)).

Thus, the uncertainties calculated by the model introduce in this paper is always larger than GUM. The systematic uncertainties in this model are extracted from the means of the Gaussian distributions that are obtained from fitting the distributions of the measured patient shifts, while the random uncertainties are extracted from the standard deviations. The GUM approach uses type A and B uncertainties, and it handles both systematic and random uncertainties in the same way by adding them by quadrature,^20^, ^21^, ^22^ which seems not to be realistic and does not agree with the statistical analysis and modeling introduced in this work. However, The CU after XC calculated by this model agrees within 0.4 mm with GUM expanded uncertainty which is obtained by arbitrary multiplication of the GUM cumulative uncertainty with a factor of 2. The major contributions to the CU after XC results from systematic uncertainties that cannot be corrected by the ExacTrac imaging guidance system such as frame, localizer, MV, and kV isocenter uncertainties. Based on the CU uncertainty calculated by this model, nearly 3.3 mm margin is recommended in the planning target volumes for SRS/SRT treatments with the frame less Brainlab system using ExacTrac for image guidance and patient setup. This approach produces more realistic uncertainties than the GUM or AAPM TG‐138^20^, ^21^, ^22^ approach based on statistical modeling and data analysis of uncertainties measured directly from the process as represented here with frameless SRS/SRT treatment.

This statistical model introduces positioning uncertainties of the different major software and hardware components in the frameless ExacTrac system. The advantages of this model include the ability to quantify the contribution of the individual components through the process and identify the weakest link that produces the largest uncertainty. In the SRS/SRT process with the ExacTrac system, the weakest link is the immobilization mask which can introduce both large systematic and random uncertainties. Although these uncertainties are reduced or eliminated by sequential processes, such as the X‐ray imaging with patient setup based on bone‐anatomy matching, the patient can still move within the mask during dose delivery after XC and XV. This may be corrected by online tracking of the patient position with the IR imaging system and frequent X‐ray snapshot to verify patient position based on bone‐anatomy matching.

The statistical model introduced in this study is useful in system commissioning and quality assurance procedural design. It provides different data acquisition, analysis, and modeling techniques that can be used to determine the positioning uncertainties associated with the different software and hardware components of the system, as well as cumulative uncertainty. In contrast to the end‐to‐end test quality assurance procedures, this system identifies the contribution of the different components, and highlights particularly the components that have the largest uncertainties contributions which can be fixed or reduced in the specific clinical application. Furthermore, this approach may be useful in failure mode and effect analysis used by forthcoming AAPM Task Group‐100,^24^, ^25^ in which the procedure is broken into different processes and each process is divided into subprocesses where the failure modes and large uncertainty sources can be identified and quantified. Once a process or subprocess with frequent failure modes or large errors is identified, integrated quality control procedures can be implemented whereby more frequent checking on this particular process is performed to achieve accurate and safe operation.

The limitation of this statistical approach is not considering many process components that are integrated together and depended on each other where their uncertainties propagate in the process and become difficult to separate. Certain components induce uncertainties at different stages of the SRS/SRT treatment process. For example, CT slice thickness introduces uncertainties at localization in the treatment planning which affects the accuracy of the stereotactic coordinate system and the positioning of isocenter. It also affects image quality of the DRRs that is used in image‐guided radiation therapy based on bone‐anatomy matching with X‐ray imaging. However, this approach can be used to develop quality control procedures that check the uncertainty of the system software and hardware component such as the example investigated here for the SRS/SRT treatment with Brainlab.

## V. CONCLUSIONS

In this work, patient setup accuracy of the frameless 6D Brainlab ExacTrac system is evaluated. Six‐dimensional translational and rotational shifts for 35 patients with mostly cranial lesions for a total of 49 total lesions treated in 1, 3, 5 fractions are investigated. These patients are simulated with the frameless head‐and‐neck mask, localized with the Brainlab localizer and planned with the iPlan treatment planning system from Brainlab. A statistical model is developed to separate and quantify the systematic and random uncertainties induced by the different hardware and software components of the 6D ExacTrac system that include the mask, localizer, IR frame, X‐ray imaging, couch, and MV and kV isocentricity. The model provides a quantitative approach to calculate the cumulative uncertainty for each patient treated with SRS/SRT treatment with the Brainlab system. This approach produces more realistic uncertainties than the GUM or AAPM TG‐138 approach based on statistical modeling and data analysis of uncertainties measured directly from the process as represented here with frameless SRS/SRT treatment. It can be used to quantify random and systematic uncertainties of the different subprocesses associated with a certain clinical producer and identify the weak links with major uncertainty contributions. Furthermore, the cumulative uncertainty from these subprocesses or hardware and software components can be calculated, which represents end‐to‐end geometric or dosimetric uncertainty of a certain clinical procedure.

## COPYRIGHT

This work is licensed under a Creative Commons Attribution 4.0 International License.

## References

[1] Schell MC . Stereotactic radiosurgery. Melville, NY: AIP; 1995.

[2] Lightstone AW , Benedict SH , Bova FJ , et al. Intracranial stereotactic positioning systems: report of the American Association of Physicists in Medicine Radiation Therapy Committee Task Group No. 68. Med Phys. 2005;32(7):2380–98. 10.1118/1.1945347 16121596

[3] Engelsman M , Rosenthal SJ , Michaud SL , et al. Intra‐ and interfractional patient motion for a variety of immobilization devices. Med Phys. 2005;32(11):3468–74. 16372417 10.1118/1.2089507

[4] Salter BJ , Fuss M , Vollmer DG , et al. The TALON removable head frame system for stereotactic radiosurgery/radiotherapy: measurement of the repositioning accuracy. Int J Radiat Oncol Biol Phys. 2001;51(2):555–62. 11567832 10.1016/s0360-3016(01)01670-4

[5] Kooy HM , Dunbar SF , Tarbell NJ , et al. Adaptation and verification of the relocatable Gill‐Thomas‐Cosman frame in stereotactic radiotherapy. Int J Radiat Oncol Biol Phys. 1994;30(3):685–91. 7928501 10.1016/0360-3016(92)90956-i

[6] van Santvoort J , Wiggenraad R , Bos P . Positioning accuracy in stereotactic radiotherapy using a mask system with added vacuum mouth piece and stereoscopic x‐ray positioning. Int J Radiat Oncol Biol Phys. 2008;72(1):261–67. 18722276 10.1016/j.ijrobp.2008.05.006

[7] Otto K and Fallone FBG . Frame slippage verification in stereotactic radiosurgery. Int J Radiat Oncol Biol Phys. 1998;41(1):199–205. 9588934 10.1016/s0360-3016(98)00005-4

[8] Breneman JC , Steinmetz R , Smith A , Lamba M , Warnick RE . Frameless image‐guided intracranial stereotactic radiosurgery: clinical outcomes for brain metastases. Int J Radiat Oncol Biol Phys. 2009;74(3):702–06. 19231101 10.1016/j.ijrobp.2008.11.015

[9] Cerviño LI , Detorie N , Taylor M , et al. Initial clinical experience with a frameless and maskless stereotactic radiosurgery treatment. Pract Radiat Oncol. 2012;2(1):54–62. 24674037 10.1016/j.prro.2011.04.005

[10] Liao HI , Wang CC , Wei KC , et al. Fractionated stereotactic radiosurgery using the Novalis system for the management of pituitary adenomas close to the optic apparatus. J Clin Neurosci. 2014;21(1):111–15. 24084193 10.1016/j.jocn.2013.03.024

[11] Muacevic A , Kufeld M , Wowra B , Kreth FW , Tonn JC . Feasibility, safety, and outcome of frameless image‐guided robotic radiosurgery for brain metastases. J Neurooncol. 2010;97(2):267–74. 19802718 10.1007/s11060-009-0023-1

[12] Murphy MJ . Intrafraction geometric uncertainties in frameless image‐guided radiosurgery. Int J Radiat Oncol Biol Phys. 2009;73(5):1364–68. 19084349 10.1016/j.ijrobp.2008.06.1921

[13] Ryken TC , Meeks SL , Pennington EC , et al. Initial clinical experience with frameless stereotactic radiosurgery: analysis of accuracy and feasibility. Int J Radiat Oncol Biol Phys. 2001;51(4):1152–58. 11704340 10.1016/s0360-3016(01)01756-4

[14] Bel A , Keus R , Vijlbrief RE , Lebesque JV . Setup deviations in wedged pair irradiation of parotid gland and tonsillar tumors, measured with an electronic portal imaging device. Radiother Oncol. 1995;37(2):153–59. 8747940 10.1016/0167-8140(95)01627-s

[15] Bel A , van Herk M , Bartelink H , Lebesque JV . A verification procedure to improve patient set‐up accuracy using portal images. Radiother Oncol. 1993;29(2):253–60. 8310153 10.1016/0167-8140(93)90255-7

[16] Jaffray DA , Siewerdsen JH , Wong JW , Martinez AA . Flat‐panel cone‐beam computed tomography for image‐guided radiation therapy. Int J Radiat Oncol Biol Phys. 2002;53(5):1337–49. 12128137 10.1016/s0360-3016(02)02884-5

[17] Jaffray DA and Siewerdsen JH . Cone‐beam computed tomography with a flat‐panel imager: initial performance characterization. Med Phys. 2000;27(6):1311–23. 10902561 10.1118/1.599009

[18] Stanley DN , Papanikolaou N , Gutierrez AN . An evaluation of the stability of image‐quality parameters of Varian on‐board imaging (OBI) and EPID imaging systems. J Appl Clin Med Phys. 2015;16(2):5088. 26103178 10.1120/jacmp.v16i2.5088 PMC5690094

[19] Verbakel WF , Lagerwaard FJ , Verduin AJE , Heukelom S , Slotman BJ , Cuijpers JP . The accuracy of frameless stereotactic intracranial radiosurgery. Radiother Oncol. 2010;97(3):390–94. 21047692 10.1016/j.radonc.2010.06.012

[20] Taylor B and Kuyatt CE . Guidelines for evaluating and expressing the uncertainty of NIST measurement results. Gaithersburg, MD: NIST; 1994.

[21] ISO Technical Advisory Group 4. Evaluation of measurement data — guide to the expression of uncertainty in measurement. JCGM 100 series. Geneva: ISO; 2008.

[22] DeWerd LA , Ibbott GS , Meigooni AS , et al. A dosimetric uncertainty analysis for photon‐emitting brachytherapy sources: Report of AAPM Task Group No. 138 and GEC‐ESTRO. Med Phys. 2011;38(2):782–801. 21452716 10.1118/1.3533720 PMC3033879

[23] Agazaryan N , Tenn SE , Desalles AA , Selch MT . Image‐guided radiosurgery for spinal tumors: methods, accuracy and patient intrafraction motion. Phys Med Biol. 2008;53(6):1715–27. 18367799 10.1088/0031-9155/53/6/015

[24] Sawant A , Dieterich S , Svatos M , Keall P . Failure mode and effect analysis‐based quality assurance for dynamic MLC tracking systems. Med Phys. 2010;37(12):6466–79. 21302802 10.1118/1.3517837 PMC3016096

[25] Noel C , Santanam L , Parikh P , Mutic S . Process‐based quality management for clinical implementation of adaptive radiotherapy. Med Phys. 2014;41(8):081717. doi:081710.081118/081711.4890589 25086527 10.1118/1.4890589 PMC4119199

